# Hemp Fiber-Reinforced Polymers Composite Jacketing Technique for Sustainable and Environment-Friendly Concrete

**DOI:** 10.3390/polym16131774

**Published:** 2024-06-23

**Authors:** Panumas Saingam, Qudeer Hussain, Gritsada Sua-iam, Adnan Nawaz, Ali Ejaz

**Affiliations:** 1 Department of Civil Engineering, School of Engineering, King Mongkut’s Institute of Technology Ladkrabang, Bangkok 10520, Thailand; panumas.sa@kmitl.ac.th; 2 Civil Engineering Department, Kasem Bundit University, Bangkok 10250, Thailand; ebbadat@gmail.com; 3 Department of Civil Engineering, Faculty of Engineering, Rajamangala University of Technology Phra Nakhon (RMUTP), 1381 Pracharat Sai 1 Road, Wong Sawang, Bang Sue, Bangkok 10800, Thailand; gritsada.s@rmutp.ac.th; 4 Department of Civil Engineering, COMSATS University Islamabad, Wah Campus, Wah Cantt 47040, Pakistan; adnan.nawaz@ciitwah.edu.pk; 5 National Institute of Transportation, National University of Science and Technology, Islamabad 44000, Pakistan

**Keywords:** hemp, confinement, concrete, neural network, compressive strength, sustainable cities and communities

## Abstract

This research suggested natural hemp fiber-reinforced ropes (FRR) polymer usage to reinforce recycled aggregate square concrete columns that contain fired-clay solid brick aggregates in order to reduce the high costs associated with synthetic fiber-reinforced polymers (FRPs). A total of 24 square columns of concrete were fabricated to conduct this study. The samples were tested under a monotonic axial compression load. The variables of interest were the strength of unconfined concrete and the number of FRR layers. According to the results, the strengthened specimens demonstrated an increased compressive strength and ductility. Notably, the specimens with the smallest unconfined strength demonstrated the largest improvement in compressive strength and ductility. Particularly, the compressive strength and strain were enhanced by up to 181% and 564%, respectively. In order to predict the ultimate confined compressive stress and strain, this study investigated a number of analytical stress–strain models. A comparison of experimental and theoretical findings deduced that only a limited number of strength models resulted in close predictions, whereas an even larger scatter was observed for strain prediction. Machine learning was employed by using neural networks to predict the compressive strength. A dataset comprising 142 specimens strengthened with hemp FRP was extracted from the literature. The neural network was trained on the extracted dataset, and its performance was evaluated for the experimental results of this study, which demonstrated a close agreement.

## 1. Introduction

The tremendous growth in population over the recent years caused a surge in construction work, which in turn caused the natural resources to be depleted [1]. It is not cost effective to import aggregates into areas where there is a scarcity of high-quality rocks or gravel. Due to the low availability of good natural aggregates, construction waste needs to be reused [2]. As a popular building element, bricks have long been a vital part of low-rise residential and commercial buildings. Additionally, clay bricks are commonly used in boundary wall construction. According to surveys conducted by highly developed countries like China and in the European Union, demolition waste is mostly brick waste. Annually, the Chinese construction industry is said to produce roughly 15.5 million tons of waste, including bricks and concrete, whereas another survey by the European Union in 2011 found that it generates construction waste of over 1 billion tons, which largely consists of brick waste [3,4]. The construction of reinforced concrete structures frequently utilizes recycled aggregated concrete (RAC) because of its properties, such as being cost-effective, sustainable, and environment-friendly [5,6]. In development processes that are sustainable, the preservation of the environment, energy efficiency, and the reduction of the use of non-renewable resources are all interconnected [3]. The destruction caused by natural calamities like frequent earthquakes generates tons of construction waste. To meet the needs of urban development and rebuilding, older buildings had to be demolished, which resulted in the accumulation of mostly fired-clay brick waste. Reusing and recycling waste is one of the strategies to save energy in modern culture. The utilization of residual fired-clay bricks has advantageous social and economic benefits for the environment, and it is effective in minimizing the cost of site clearance and dumping [7]. Sustainable cities and communities is one of the sustainable development goals (SDGs), which supports to improve the quality of cities.

A practical and affordable solution is to utilize fired-clay solid brick waste aggregates (CBA) to make recycled aggregate concrete (RAC). In modern construction, it is used to make road bases, backfillings, and sidewalks [8]. Compared to natural aggregate concrete (NAC), RAC-CBA is not widely used in structural applications, particularly in load-bearing components, as it has inferior properties like lesser ductility, stiffness, and lower compressive strength [9]. The use of recycled aggregate is thoroughly reviewed so that it can act as a basis for future research on recycled aggregates that use waste bricks. The particle density of RAC is 5–15% less than that of NAC [10]. This is also attributed to the less dense mortar that was used to bond the recycled aggregate [10]. The compressive strength of concrete was found to be reduced by 11% and 20%, respectively, when 20% and 50% of natural aggregates were replaced with fired-clay brick aggregates, according to previously conducted research [11]. Due to their capacity to hold more water than natural aggregates, recycled aggregates have a significant impact on the mechanical attributes of recycled aggregate concrete (RAC). It is acknowledged that for high-grade RAC, the compressive strength is not significantly different from NAC [12]. Additionally, it was concluded in the previous research that when the number of NAs being replaced is lower than 30%, the difference in RAC’s and NAC’s compressive strengths is minimal [13]. One previous study conducted by González et al. [14] revealed that compressive strength can be reduced by a maximum of 28% when recycled brick aggregates are used to replace 100% of natural aggregates. When recycled brick aggregates replaced natural aggregates completely, i.e., 100%, a prior study by González et al. [14] showed that the compressive strength decreased by a maximum of 28%. Furthermore, the tensile strength of the concrete remained almost unchanged until 35% of the replacement ratio. A previous study shows similar results to that mentioned above, which exhibited a decrease in RAC compressive strength by 30% when fully replaced [7]. According to the results, higher water absorption, lower fragmentation resistance, and lower density are the inferior properties of RAC. Another study by Cachim [15] showed that concrete properties remained unaffected by a replacement ratio of 15%, whereas mechanical properties were reduced by 20% for replacement ratios of 30%. In a prior study by Medina et al. [16], the compression strength decreased by up to 39% when recycled aggregates replaced the natural aggregates by 40%. A similar previously conducted study on recycled concrete [17] came to the conclusion that a maximum (30% replacement ratio) of NAs must be applied for structural applications in order to preserve the proper strength and stiffness. Recycled brick aggregates have reduced mechanical properties, but they have some advantages, like being comparatively lightweight (brick has a lower density than stone) and having improved fire resistance (brick has high refractory properties) [18]. In contrast to concrete manufactured with natural aggregate, recycled brick aggregate concrete possesses fewer favorable qualities, according to the studies indicated in the preceding paragraphs. However, the difference is insignificant at 30% or less than the 30% replacement ratio.

Strengthening RAC-CBA with synthetic or natural fiber-reinforced polymer (FRP) sheets is a popular method of reducing their subpar compressive strength. Synthetic FRPs are frequently used in projects aimed at strengthening structures [15,19] and enhancing RACs’ structural qualities [20,21], but they are also costly [22]. In addition, the chemicals employed in the manufacture of these synthetic FRPs may expose sensitive individuals to skin conditions such as allergies and contact dermatitis. Recently, natural fibers have been proposed as a possible substitute for synthetic fibers [23]. Natural FRPs are considerably cheaper and do not increase the risk of skin diseases than synthesized FRPs [24,25]. However, natural FRPs had a variety of drawbacks, such as increased moisture absorption, lower fire resistance, weaker mechanical qualities, and less durability. Due to several environmental issues, researchers are inspired to conduct further in-depth studies to look for materials that are sustainable. More environmentally friendly natural fibers produced from synthetic and natural resources that have been successfully used for the repair and reinforcement of concrete include hemp, jute, cotton, sisal, flax, and fibers [26,27]. These fibers have reasonable prices, and their manufacturing generates remarkably less CO_2_. Fiber ropes have several benefits that have been emphasized, including availability, affordability, ease of usage, and being environmentally friendly. An early study [28] investigated how sisal, hemp fibers, and jute affected concrete’s ultimate compressive strength and strain. It was found that the ultimate compressive strength increased in specimens that were constrained by hemp fiber ropes. It was demonstrated in a different study that hemp ropes greatly reduced the bending strain and deflection of beams [29]. In a previously conducted study [30], concrete columns were wrapped in three layers of hemp ropes. Numerous ratios were considered for column slenderness. The ductility and axial strength of constrained columns were higher than those of their corresponding reference ones.

## 2. Research Significance

This study is significant because it aims to bolster the mechanical properties of concrete by incorporating recycled fired-clay solid brick aggregates at a 50% replacement ratio. It introduces a novel approach by utilizing square-shaped specimens constructed with these recycled aggregates to evaluate the effectiveness of hemp fiber-reinforced ropes (FRR). The investigation delves into parameters like the number of exterior layers of hemp fiber and the unconfined concrete strength. Additionally, it employs statistical analysis to compare stress and strain results with existing models. Moreover, the integration of artificial neural networks (ANN) for predicting compressive strength adds a cutting-edge dimension to the research, enhancing its potential impact on future construction practices.

## 3. Experimental Program

### 3.1. Test Matrix

**Figure 1:**
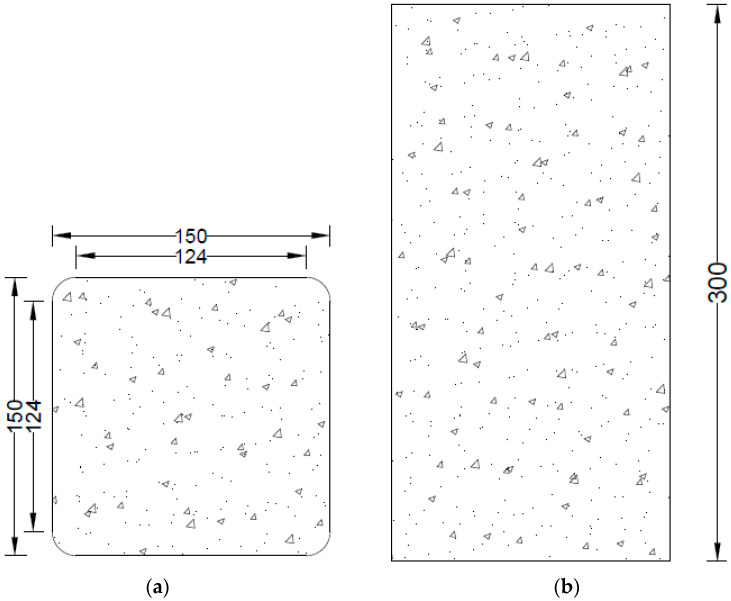
Detail of square specimen having 13 mm radius: (**a**) top view showing the width and corner radius; (**b**) longitudinal view representing the height of the square specimen.

In this research work, three different groups of concrete were made depending on their strength (low-strength, medium-strength, and high-strength). Each group has eight square specimens. All 24 square samples were cast. The square specimens have dimensions of 150 mm in width and 300 mm in height. Previously, it was observed that the confined square specimens without rounded corners failed due to the sharp edges [16]. Therefore, to avoid this type of failure, square specimens were rounded off to attain a radius (R) of 13 mm to avoid premature rupture of hemp fiber ropes at the sharp edges, as shown in Figure 1. In each group of concrete, there were two control specimens without the hemp fiber ropes, and single, double, and triple layers of hemp fiber ropes were applied separately on two specimens. Hence, there are four different types of specimens in three groups of concrete (i.e., low-strength, medium-strength, and high-strength groups). The average result of two specimens was taken in this research due to the high construction cost of these kinds of specimens at the laboratory level. Tested specimens’ designations and their strengthening detail are given in Table 1. SR13 is used for square specimens having a 13 mm corner radius, and LS, MS, and HS are for low-, medium-, and high-strength concrete, respectively. CBA is used for fired-clay solid brick aggregate waste, CON is for control specimens, while 1H, 2H, and 3H are used for one, two, and three layers of hemp fiber ropes, respectively. It is noteworthy that this study did not include specimens constructed with natural aggregates. This is because the efficacy of hemp FRR has already been addressed on natural aggregate concrete in the work by Hussain et al. [28]. Hussain et al. [31] tested the efficacy of hemp FRR on square specimens by varying corner radii and reported that the efficacy of hemp FRR was proportional to the corner radii provided.

### 3.2. Properties of Materials

**Figure 2:**
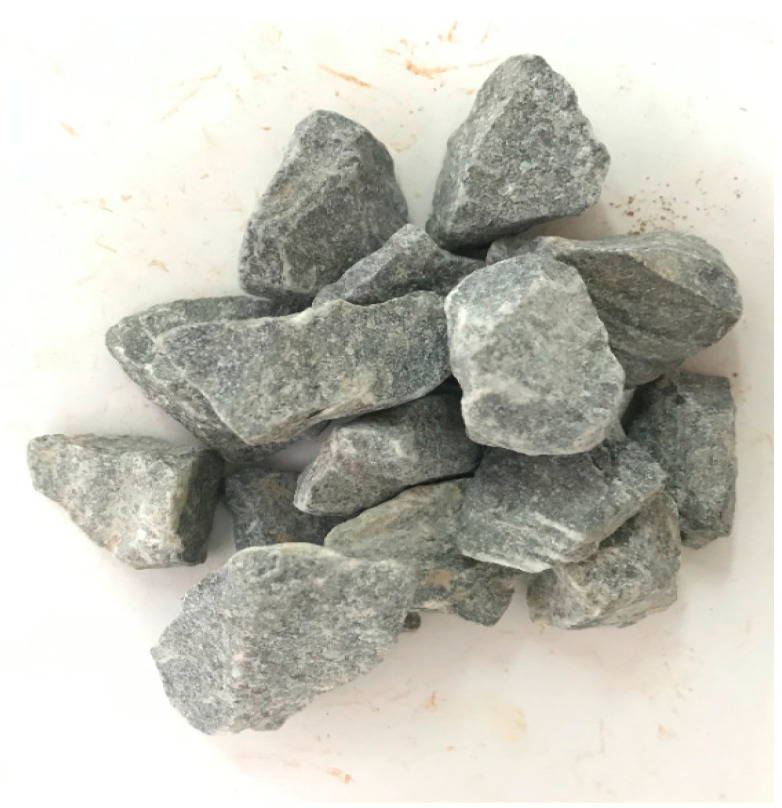
Natural aggregate.

**Figure 3:**
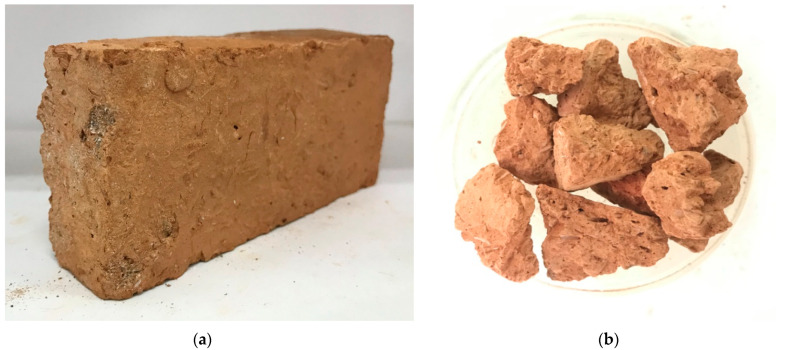
(**a**) Fired-clay solid brick; (**b**) aggregate obtained from fired-clay solid bricks.

Eight square specimens were made for each low-strength, medium-strength, and high-strength concrete group, respectively. Portland cement Type-1 was used as a binder. In each specimen, natural aggregates were partially replaced with fired-clay solid bricks waste coarse aggregates. An amount of 50% replacement of fired-clay solid brick waste aggregates (CBA) was used for natural coarse aggregates in low-strength, medium-strength, as well as in high-strength concrete specimens. In this study, the percentage of replacement measured was in mass units. Figure 2 exhibits the natural coarse aggregate employed in this study. Figure 3a shows the fired-clay solid brick used for waste coarse aggregate production, whereas Figure 3b represents the crushed fired-clay solid brick coarse aggregate with a maximum size of 25 mm.

The mix design of concrete for making low-strength, medium-strength, and high-strength concrete is given in Table 2. The quantities presented in Table 2 are the required quantities in kilograms to achieve the target strength of low, medium, and high-strength concrete. The first column defines the type of material, and the corresponding columns in the same row present quantities required in kilograms for one cubic meter of concrete. Some important properties of fired-clay solid bricks, like compressive strength, water absorption, and density, were determined according to ASTM standards [32,33]. Results concluded that these bricks have a compressive strength of 6.26 MPa, 12.3% water absorption, and 145 kg/m^3^ density.

**Figure 4:**
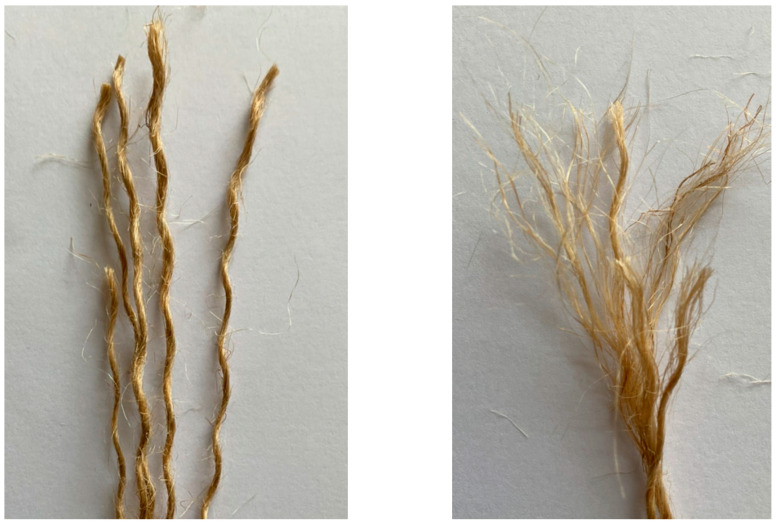
Hemp fiber used in this research work.

The hemp fiber ropes were fully fixed with the samples by applying two-part epoxy. It was made by mixing resin and hardener in a 2:1 ratio. This epoxy was applied using a brush to fix the fibers with the square specimens. This epoxy has a tensile strength of 50 MPa and flexural strength of 75 MPa, while its percent elongation was 2.5%. Important tensile properties of hemp fiber ropes were calculated using ASTM A931-18 [34] and ASTM E8/E8M-13 [35]. A loading rate of 1.5 mm/min was applied during tensile testing of hemp fiber ropes. Tensile coupons were utilized to determine the mechanical properties. Table 3 shows the properties of hemp fiber ropes, and Figure 4 shows the hemp fiber rope used in this research work.

### 3.3. Construction of Square Specimens and Strengthening Process

**Figure 5:**
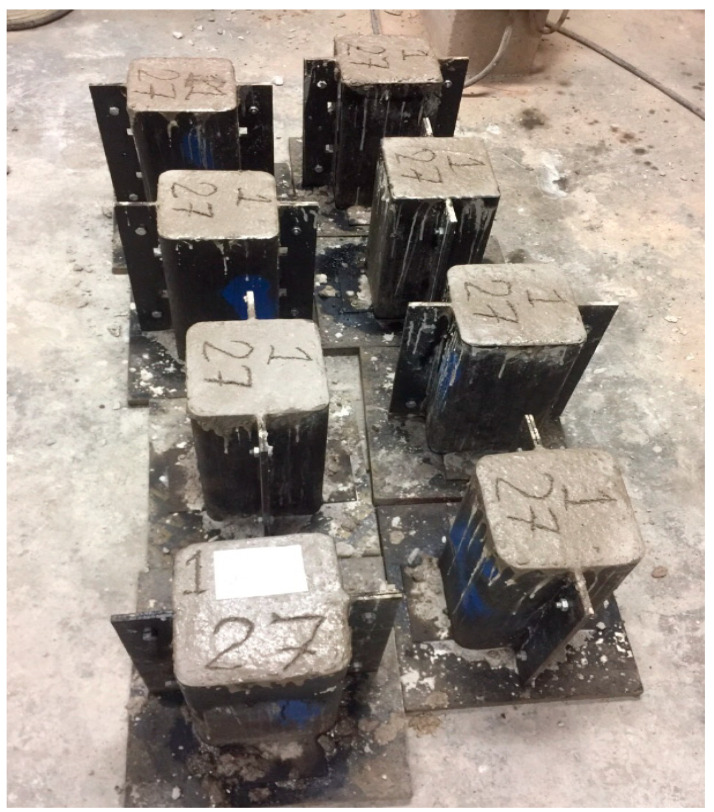
Construction of square specimens having 13 mm rounded corners.

**Figure 6:**
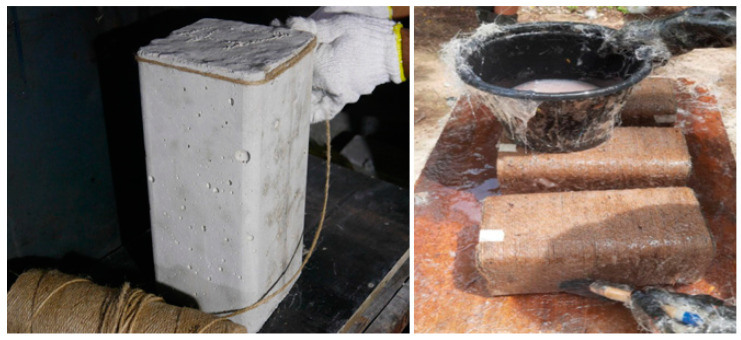
Application of two-part epoxy and fiber ropes to square specimens.

Square specimens were prepared in specially designed square molds with a 13 mm radius in the corners, as can be seen in Figure 5. After making the square specimens, these specimens were water-cured for 28 days. After the curing period, the specimens were strengthened using hemp fiber ropes. In prior research [28], it was observed that hemp fiber ropes have a flat plateau of the stress–strain portion after reaching 10 MPa of strength, and then it changes to a sharp curve. Therefore, it is important to give a pretension strength of 10 MPa during the strengthening process, and this was achieved by developing the same mechanical setup, as suggested in previous research on hemp fiber ropes [28]. During the wrapping of hemp rope on square specimens, one end was attached to the specimen with the help of super glue, as shown in Figure 6 (left), and then the wrapping of hemp ropes was carried out from top to bottom of the specimen in such a way that there was no gap between the ropes. After that, the end of the rope was attached to the bottom end of the specimen again with the help of epoxy. After this step, epoxy was applied to the specimen in such a way that the hemp fiber ropes and the concrete surface were fully saturated with epoxy, as exhibited in Figure 6 (right). For specimens having two layers of hemp ropes, the first layer was applied similarly to the method described above, and samples were allowed to dry for 12 h. Then, the second layer of hemp fiber rope was applied. Similarly, for three layered specimens, after the application of the second layer, a gap of 12 h was given, and then the third layer of hemp fiber ropes was applied. It is important to mention that special care was given during the application of the second and third layers of ropes, which are applied exactly above the first and second layers, respectively. It must be acknowledged that the hemp ropes were positioned similarly to the layers under this layer in terms of application.

### 3.4. Instrumentation and Testing Setup

**Figure 7:**
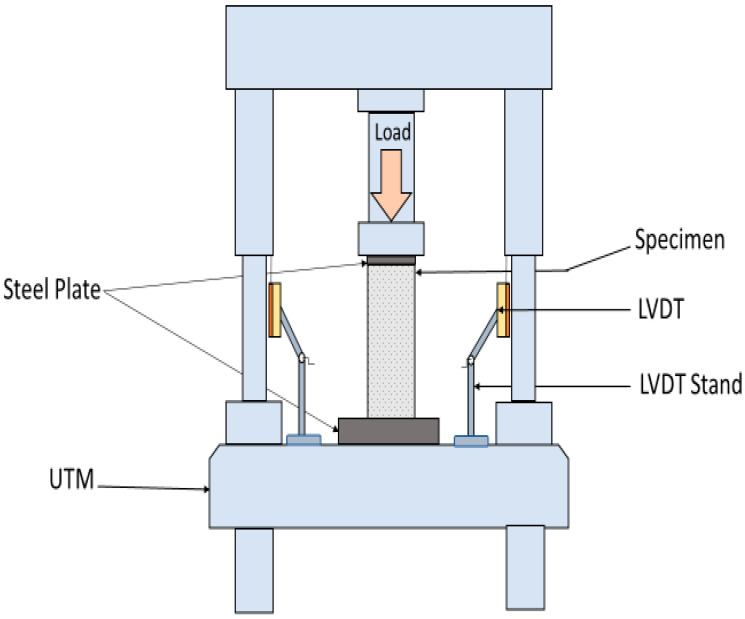
Mechanical testing setup.

The compressive test was conducted using a Universal Testing Machine (UTM), which had a maximum load capacity of 2 MN. A 4000 N/sec loading rate was used during testing. For more accuracy, a calibrated load cell was also used at the top of the sample to measure the load. Axial deformation of specimens was recorded during the test using three linear variable displacement transducers (LVDTs) affixed 120° apart from each other around the samples. Steel plates were also placed on the top and bottom of the specimens to prevent unintentional weight transfer to the hemp fiber ropes during large axial deformations. Figure 7 shows the mechanical testing setup used in this research work.

## 4. Experimental Results

### 4.1. Axial Stress–Strain Response

Compressive strength and percent strain values are listed in Table 4 for all tested samples. Results of Table 4 summarized that the peak stress for low-strength concrete increased by 59%, 117%, and 181% for single, double, and triple layers of hemp rope confinement, respectively and their strain increment for a single, double, and triple layer of confinement was 104%, 438%, and 564%, respectively. The stress and strain of medium- and high-strength concrete were likewise increased by hemp ropes. Likewise, there was a notable escalation in peak stress observed across medium- and high-strength concrete specimens under 1-, 2-, and 3-layer confinements, registering increments of 37%, 79%, and 114%, as well as 32%, 65%, and 94%, respectively. Furthermore, the strain augmentation for single, double, and triple layers of confinement exhibited substantial rises of 98%, 256%, and 400%, and 94%, 131%, and 192%, respectively.

**Figure 8:**
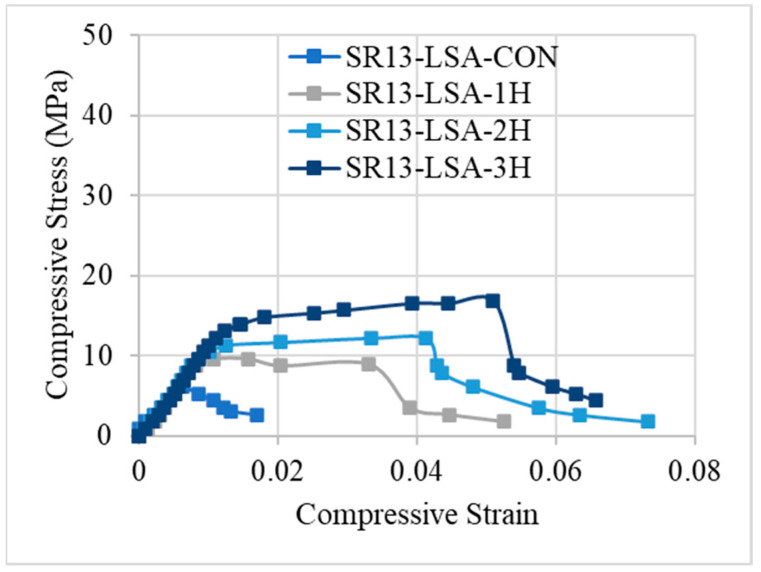
Stress–strain response of low-strength concrete with and without confinement.

**Figure 9:**
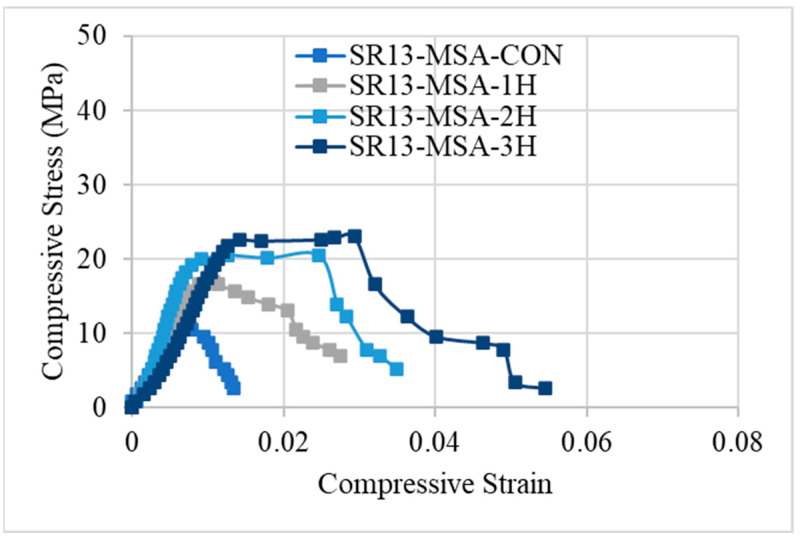
Stress–strain response of medium-strength concrete with and without confinement.

**Figure 10:**
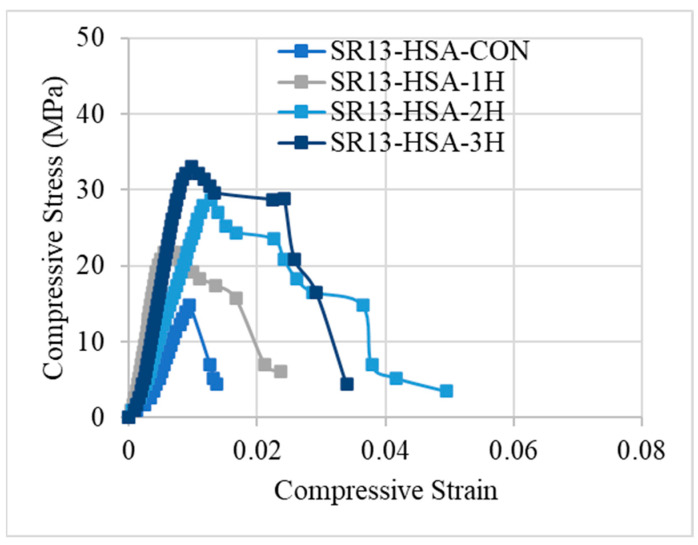
Stress–strain response of high-strength concrete with and without confinement.

Stress–strain responses of low-, medium-, and high-strength concrete specimens with and without confinement are illustrated in Figure 8, Figure 9 and Figure 10, respectively. From the results, it can be concluded that controlled specimens of all three types of concrete strength exhibited a brittle failure pattern as their strength abruptly decreased after reaching their maximum level. However, the findings of confinement showed that hemp fiber rope increases the ductility for all three types of concrete strength. For confinement, generally, three trends of stress–strain responses were observed for confinement configurations. Type 1: after the first peak, strain decreases gradually. Type 2: typical bilinear stress–strain response. Type 3: bilinear stress–strain response, but having reduced slope as compared to type 2 behavior. It is noted that Joyklad et al. [36] tested natural and recycled brick aggregate concrete under axial compression. A reduction of 37.0% and 58.6% in compressive strength was reported, corresponding to the replacement of 50% and 100% natural coarse aggregates with recycled brick aggregates. It is noted from this study that the minimum enhancement in compressive strength by using a single proposed hemp FRR confinement was 32%, whereas it reached up to 181% in the case of a three-layer hemp confinement. In light of these findings, it is suggested that the proposed hemp FRR confinement can restore the strength of the recycled brick aggregate to an extent that matches or surpasses the corresponding strength of the natural aggregate concrete (i.e., without the replacement of natural coarse aggregates).

Figure 8 demonstrates the stress–strain response of hemp rope-confined specimens for low-strength concrete. Results revealed that type-1 behavior was observed for confinement in a single layer, with a progressive decline in stress–strain following the first peak. This response varies as the number of hemp rope layers increases. For double and triple layers of hemp rope confinement of low-strength concrete, both showed a type-2 response, i.e., bilinear stress–strain response. After achieving the first peak, the stress–strain increases gradually and then drops suddenly after the second peak. A similar bilinear curve was also observed in the previous research work [17] on recycled brick aggregate concrete confined by FRP. Additionally, the graph demonstrates that it is difficult to distinguish between the first slopes of all the confined specimens. It should be mentioned that the ultimate strength and strain increase as the number of hemp rope layers increases.

The stress–strain response of medium-strength concrete specimens is shown in Figure 9. Similar to low-strength concrete, single-layer confinement in medium-strength concrete also exhibited type-1 behavior, i.e., after reaching the first peak, the strength decreases gradually. However, for double and triple layers of hemp ropes, medium-strength concrete showed type-3 behavior, unlike low-strength concrete. The slope of the bilinear portion is reduced as compared to the typical bilinear curve. The stress–strain response for high-strength hemp rope-confined concrete is different from the other two concrete types, as illustrated by Figure 10. The stress–strain behavior was type-1 for single, double, and triple layers of confinement. After achieving the first peak, stress–strain steadily falls until failure. Overall, it can be deduced that hemp rope fibers are effective in enhancing the strength and ductility of all three types of concrete. Stress–strain behavior also revealed a crucial point: as strength increased, the behavior changed from usual bilinear (type-2) to type-3 and eventually type-1 when strength further increased.

### 4.2. Hemp Rope Layers Effect on Low-, Medium-, and High-Strength Concrete

**Figure 11:**
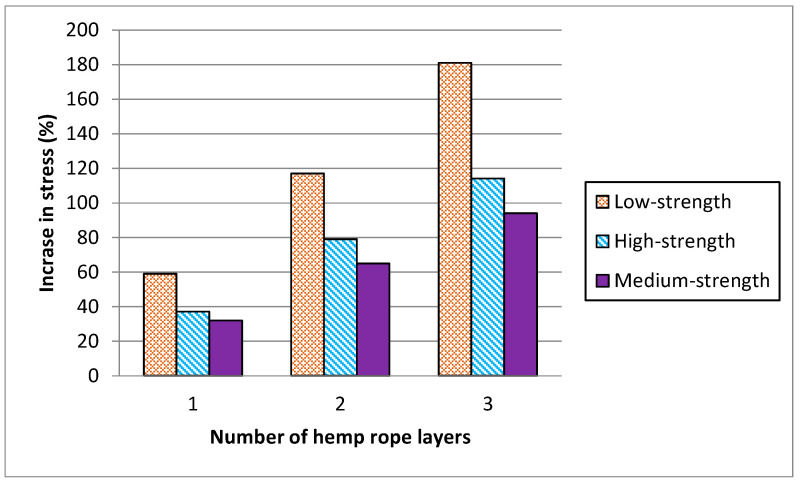
Comparison of increase in peak stress as a function of number of hemp ropes.

**Figure 12:**
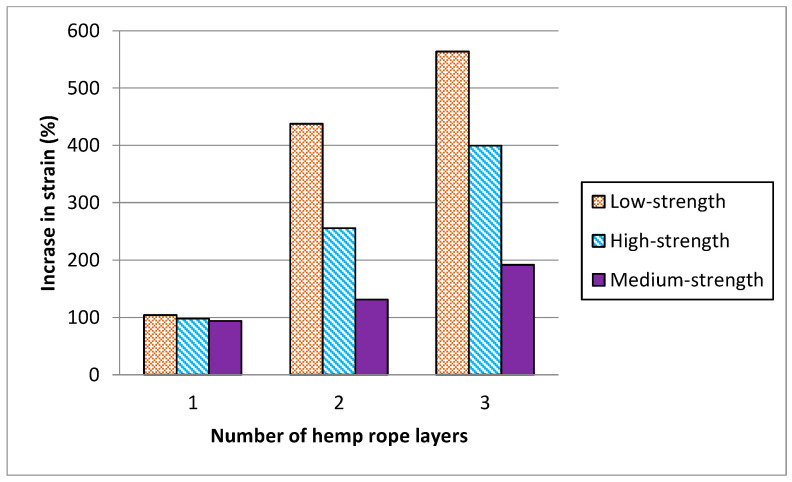
Comparison of increase in strain corresponding to ultimate compressive strength due to number of hemp ropes.

Figure 11 and Figure 12 represent the comparison in increase between peak stress and the corresponding strain of low-, medium-, and high-strength concrete specimens with hemp fiber rope confinement. Results revealed that as the concrete strength increased, the strength and strain increment decreased. For single, double, and triple layers of hemp rope confinement, the peak strength of low-strength concrete specimens was higher than medium- and high-strength concrete specimens by 22%, 38%, 67%, 27%, 52%, and 87%, respectively. Similarly, the ultimate strain of low-strength concrete specimens was significantly greater than medium- and high-strength concrete specimens by 06% and 10% for a single layer, 182% and 307% for a double layer, 164% and 372% for a triple layer of hemp rope confinement, respectively. These findings confirm the previous research on confinement by natural fiber reinforcement polymers [37]. The performance of hemp rope confinement in low-strength concrete is better than in medium- and high-strength concrete. The performance of hemp ropes in medium- and high-strength concrete, however, was also superior to that of their specimens without confinement by hemp rope.

### 4.3. Failure Pattern of Specimens

**Figure 13:**
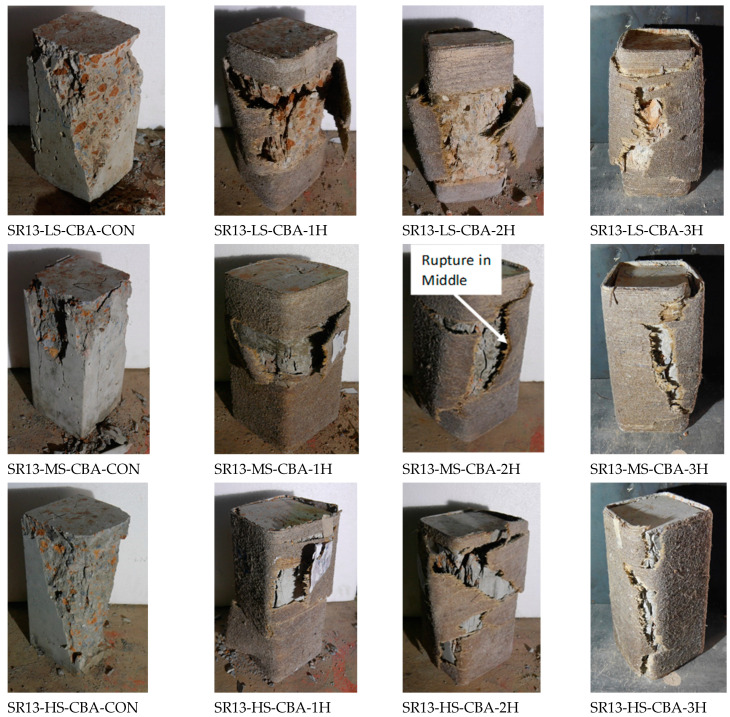
Ultimate failure pattern of low-strength, medium-strength, and high-strength square specimens with and without confinement.

Failure patterns of specimens with and without confinement are illustrated in Figure 13. Control specimens of low-, medium-, and high-strength concrete failed by the cracking and crushing of concrete through the specimen. Similar results have been reported in the literature that cracking and crushing are dominant failure modes [38]. Moreover, SR13-HS-CBA-CON, a controlled specimen, demonstrated abrupt failure during testing as compared to medium- and low-strength concrete specimens. At the same time, the hemp rope-confined specimens failed because of the hemp rope failure in the hoop direction. It was observed during testing that hemp ropes were not debonded from the specimens, which demonstrated that epoxy strength was good and maintained the bond between the concrete surface and hemp fiber ropes. Snapping sounds were heard for samples with more than one layer of hemp rope fibers, indicating the gradual fracturing of hemp ropes in underlying layers. In the literature, similar observations have previously been described [39,40]. Furthermore, it can be seen from Figure 13 that specimens with single and double layers of hemp fiber rope confinement failed to rupture the hemp rope in the middle of the specimens. On the other hand, specimens with three layers of hemp ropes also failed due to the tensile rupture of hemp rope, but their rupture spread over a wider area and up the entire height of specimens. As a result, specimens with three layers of hemp ropes were able to withstand greater stress and strain than single- and double-layer confined specimens. The same experimental outcomes have been reported in the prior mentioned literature [17,39].

## 5. Comparison with Analytical Models

### 5.1. Detail of Existing Models

In recent years, numerous researchers have worked on the models’ development to anticipate the stress–strain behavior of specimens with unidirectional FRP confinement. Different models have been proposed for the assessment of the stress and strain of concrete having FRP confinement. From the study of the literature, various models have been found for the determination of FRP-confined concrete’s compressive strength, as indicated in Table 5. A number of researchers, including Cusson and Paultre [41], Legeron and Paultre [42], Shehata et al. [43], Triantafillou et al. [44], Wang et al. [45], and Akiyama et al. [46] have proposed models which can be applied for the prediction of the stress and strain of square samples. The following equation can be used to represent almost all of the models that are being found for FRP-confined concrete:

(1) $$f_{cc}=\left[1+k_{1}\left(\frac{fl}{f_{co}}\right)\right]f_{co}\;$$

The notation $f_{cc}$ is used to represent the confined compressive strength of concrete, and *f_co_* is used for unconfined compressive strength, whereas the terms confinement effectiveness coefficient is represented by *k*_1_ and $f_{l}$ is used to represent lateral confining pressure. The following formula is used to relate the $f_{l}\;$ with the FRP thickness and strength of the square samples.

(2) $$fl=2f_{T}t\frac{ks}{D}$$

In the above equation, the tensile strength of FRP in the hoop direction is represented by $f_{T}$, FRP thickness is represented by *t*, and *D* denotes the diameter of concrete, while *k_s_* represents the shape factor, and it is described as the ratio of effective confinement area to concrete’s total cross-sectional area. ACI318 defines the shape factor by using the equation given below:

(3) $$ks=1-{\left(b-2R\right)}^{2}+\left(\frac{h-2R^{2}}{3A_{g}}\right)$$

where *A_g_* is defined as the concrete cross-sectional area, which can be calculated by the use of the following equation:

(4) $$Ag=bh-\left(4-\pi \right)R^{2}$$

The column possessing the same volumetric ratio of FRP as that of the original rectangular column is defined as the equivalent circular column. By using this definition, the equation given below could be utilized for calculating the diameter of an equivalent circular column, and in the equation given below the width and depth of the square section are represented by b and h, respectively:

(5) $$D=\frac{2bh}{b+h}$$

### 5.2. Comparison of Experimental Results with Analytical Models

**Figure 14:**
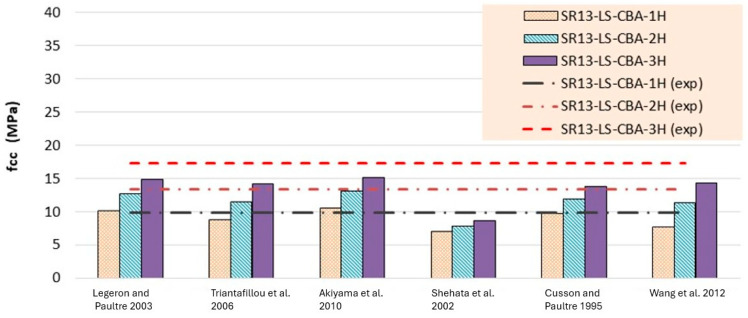
Stress result comparison of low-strength concrete group [40,41,42,43,44,45].

**Figure 15:**
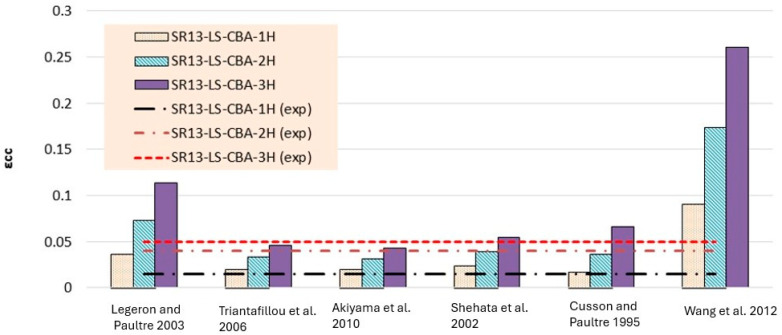
Strain result comparison of low-strength concrete group [40,41,42,43,44,45].

**Figure 16:**
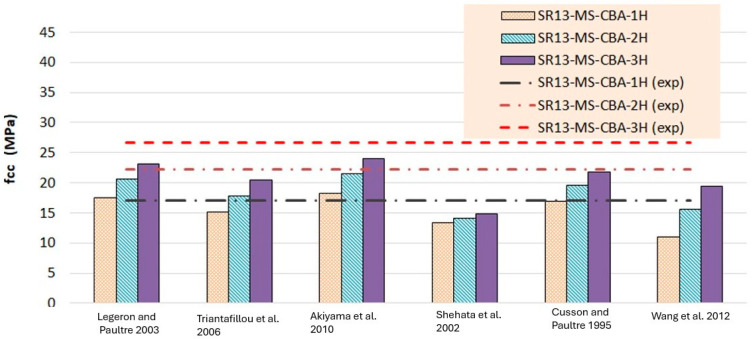
Stress result comparison of medium-strength concrete group [40,41,42,43,44,45].

**Figure 17:**
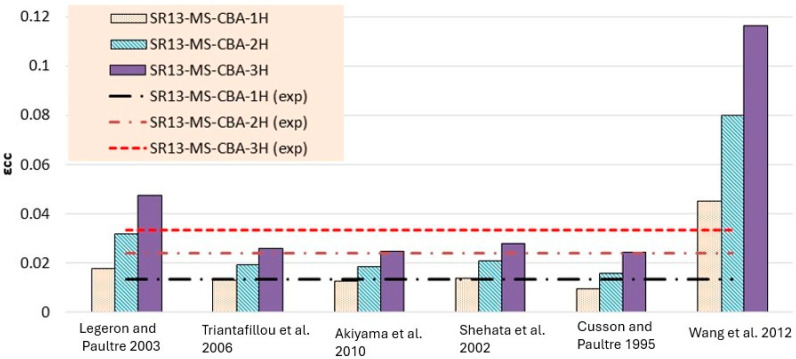
Strain result comparison of medium-strength concrete group [40,41,42,43,44,45].

**Figure 18:**
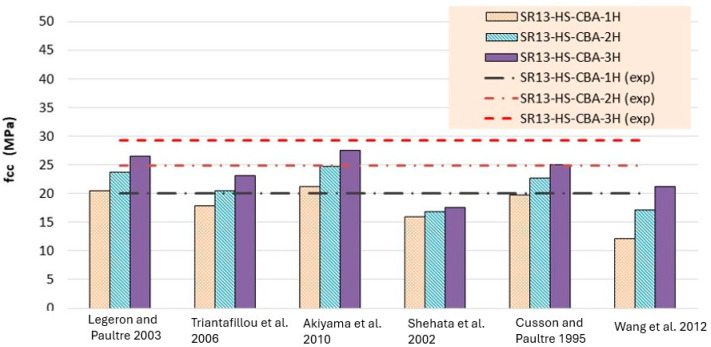
Stress result comparison of high-strength concrete group [40,41,42,43,44,45].

**Figure 19:**
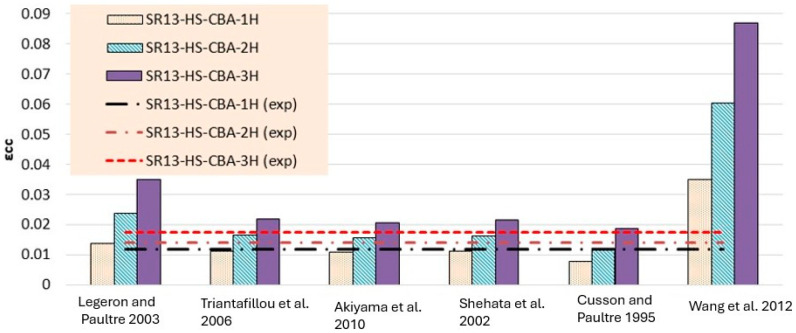
Strain result comparison of high-strength concrete group [40,41,42,43,44,45].

Experimentally determined stress and strain values for low-, medium-, and high-strength square specimens confined by hemp ropes are compared with the theoretical models listed in Table 6, Table 7 and Table 8 and presented in Figure 14, Figure 15, Figure 16, Figure 17, Figure 18 and Figure 19, respectively. From the stress results of Table 6 and Figure 14 for low-strength concrete specimens, it is evident that for the first layer of confinement of hemp ropes, the model proposed by Cusson and Paultre is in good agreement with experimental data and that most of the models are close to the experimental results. While for three-layer confinement of hemp ropes, all models underestimate the experimental results. Similarly, for the double layer of confinement, most theoretical models underestimated the experimental results, whereas the model presented by Legeron and Paultre is close to the experimental results, and Akiyama et al.’s [46] model estimate is in accordance with the experimental result.

According to the results of Table 7 and Figure 16, for medium-strength concrete specimens, some stress models for confinement with a single layer of hemp ropes overestimated the results, while others underestimated them. However, the results of models proposed by Cusson and Paultre [41] and Legeron and Paultre [42] are in good agreement with the experimental findings. For the double layer of confinement, the results of some models are lower than the experimental results, and most of the models’ results are close to the experimental results. The model proposed by Akiyama et al. [46] is roughly equivalent to the experimental results for double-layer confinement of medium-strength concrete. While for three-layer confinement of hemp ropes, the results of some of the models are near to the experimental results, most of the theoretical models underestimated the experimental results. Table 8 and Figure 18 also display the stress results for hemp rope confinement of high-strength concrete square specimens. Models proposed by Legeron and Paultre [42] and Akiyama et al. [46] are approximately equivalent to the experimental results for all three types of confinement configurations. Cusson and Paultre’s [41] model result for a single layer of confinement is similar to the experimental result. Results of all other models highly underestimated the finding for all three types of confinement configurations.

Theoretical results of strain for low-strength concrete in Table 6 and Figure 15 concluded that most of the models overestimated the results. Models proposed by Legeron and Paultre [42], Cusson and Paultre [41], and Wang et al. [45] give highly overestimated values of theoretical results for single, double, and triple layers of hemp rope confinement. The results of models presented by Triantafillou et al. [44], Akiyama et al. [46], and Shehata et al. [43] estimated the strain values accurately for triple-layer confinement. For medium-strength concrete, the majority of the models’ results are comparable to the experimental results for all three types of confinement configurations, as can be seen in Table 7 and Figure 17. The model proposed by Cusson and Paultre [41] underestimated the results, whereas the results of models given by Legeron and Paultre [42] and Wang et al. [45] significantly overestimated the values of theoretical results for single, double, and triple layers of hemp rope confinement. The results of models provided by Triantafillou et al. [44], Akiyama et al. [46], and Shehata et al. [43] estimated the strain values accurately for all types of confinement configuration, particularly for the first layer of confinement results, which are very close to the experimental values.

For high-strength concrete, the strain outcomes depicted in Table 8 and Figure 19 exhibit a variation similar to that observed in medium-strength concrete. The model proposed by Cusson and Paultre [41] consistently underestimates the results, while those presented by Legeron and Paultre [42] and Wang et al. [45] tend to overestimate the experimental values across single, double, and triple layers of hemp rope confinement. Conversely, the models outlined by Triantafillou et al. [44], Akiyama et al. [46], and Shehata et al. [43] accurately predict strain values for all types of confinement configurations, particularly for the initial layer of confinement, aligning closely with experimental data. However, there is a paucity of models tailored specifically for square specimens, particularly those utilizing hemp rope confinement, with existing models primarily geared towards CFRP confinement in concrete. Thus, further research is imperative to comprehensively grasp the effects of hemp rope confinement on square specimens.

## 6. Strength Prediction by Artificial Neural Network

**Figure 20:**
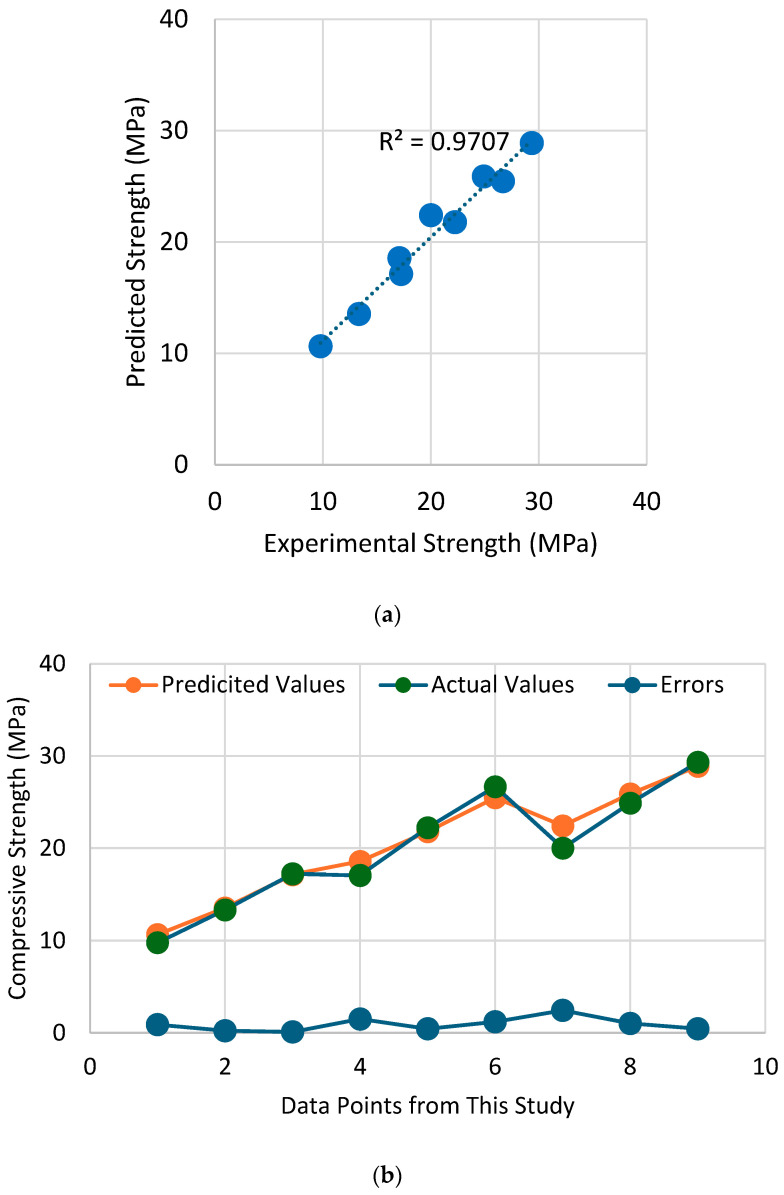
Performance of ANN model in predicting the compressive strength of hemp-confined concrete (**a**) comparison between experimental and predicted strengths and (**b**) error in compressive strength prediction.

In the past, AI techniques have successfully been utilized to predict the compressive strength of concrete under external passive confinement. Naderpour et al. [47] employed artificial neural networks (ANN) to forecast the compressive strength of FRP-confined concrete, which yielded excellent efficiency. Cascardi et al. [48] found that the prediction of compressive strength of FRP-confined concrete by ANN models was superior to the prediction by existing analytical expressions. Ahmad et al. [49] predicted the compressive strength of recycled aggregate concrete by using ANN models and reported comparable efficiency with the prediction by Gene Expression Programming. The present study also utilized the ANN model to predict the compressive strength of hemp-confined concrete. For this purpose, a dataset comprising 142 concrete specimens confined with hemp ropes was taken from the literature. The details of this dataset are presented in Appendix A. The compressive strength of confined concrete is related to the compressive strength of unconfined concrete, the number and diameter of hemp ropes, and the size of the specimen. Moreover, the shape of the cross-section has been found to impact the confinement efficiency of external confinement. For rectilinear sections, the corner radius plays an important role in determining the efficacy of external confinement. Therefore, the explanatory variables included the shape of the cross-section, the thickness of the ropes, the tensile strength of the ropes, and the strength of unconfined concrete. Utilizing Visual Studio Code (VS Code) as the Integrated Development Environment (IDE), machine learning models were crafted and executed. The models were constructed leveraging the sci-kit-learn (sklearn) library within Python. Employing the train_test_split function from sci-kit-learn, the dataset underwent partitioning into training and testing subsets, with an 80/20 ratio allocation. Additionally, the creation of the neural network model entailed the utilization of the Keras library integrated within TensorFlow. The prediction of the ANN model is shown in Figure 20. The coefficient of determination $R^{2}$ was used to evaluate the performance. It is vital to note that an $R^{2}$ value of 0.97 suggests an excellent performance of the ANN model in this matter. Moreover, the error between experimental and predicted strengths shown in Figure 20b is close to zero.

## 7. Discussion

The aim of this project is to enhance the mechanical properties of RAC square specimens that incorporate 50% waste-fired-clay solid brick aggregates (CBA) in replacement of natural aggregates. Moreover, the confinement effect on three different strengths of concrete was also investigated. An experimental framework was conducted to achieve the objectives of this research work. The following are the important conclusions drawn from this research work:

1. All Control specimens (low, medium, and high) strength concrete exhibited normal compression failure by cracking and crushing of concrete through the specimens. The stress–strain response of control specimens showed a brittle failure pattern for all three types of concrete strength.

2. Hemp rope confinement exhibited significant improvement in strength as well as in ductility of square specimens. Peak strength and the corresponding strain of concrete increased with the increase in hemp rope layer numbers. The stress–strain behavior of hemp rope confinement showed three different types of stress–strain behavior, including the typical bilinear behavior of FRP confinement.

3. Low-strength concrete specimens with hemp ropes’ similar configurations exhibited improved effectiveness in enhancing peak strength and the corresponding strain as compared to the medium- and high-strength concrete specimens.

4. Comparison of experimental and theoretical findings deduced that only a limited number of strength models were accurate, i.e., the models by Legeron and Paultre [40] and Akiyama et al. [46], while the majority were not. This can be attributed to the different nature of FRPs for those models. A more thorough investigation is needed for specimens that are restrained by double and triple layers of hemp rope because the existing strain models are highly accurate only for confinement by a single layer of hemp rope.

5. A machine learning algorithm, i.e., artificial neural network (ANN), was adopted to predict the compressive strength of hemp-confined concrete. The training of the ANN model was performed on an existing dataset of 142 hemp-confined specimens, whereas the prediction was compared with the experimental results of this study. The model demonstrated an excellent efficiency, yielding an $R^{2}$ value of 0.97. This highlights the efficacy of the ANN model in predicting the compressive strength of hemp-confined concrete.

## Figures and Tables

**Table 1:** Specimens designations and their strengthening detail.

Group	Specimen Name	Replacement Level of CBA	No. of Layer	No. of Specimen
Group 1	SR13-LS-CBA-CON	50%	None	2
	SR13-LS-CBA-1H	50%	1	2
	SR13-LS-CBA-2H	50%	2	2
	SR13-LS-CBA-3H	50%	3	2
Group 1	SR13-MS-CBA-CON	50%	None	2
	SR13-MS-CBA-1H	50%	1	2
	SR13-MS-CBA-2H	50%	2	2
	SR13-MS-CBA-3H	50%	3	2
Group 3	SR13-HS-CBA-CON	50%	None	2
	SR13-HS-CBA-1H	50%	1	2
	SR13-HS-CBA-2H	50%	2	2
	SR13-HS-CBA-3H	50%	3	2

**Table 2:** Mix design for different groups of concrete.

Mix Material	Quantity Required in kg for 1 m^3^ of Concrete		
	Low Strength	Medium Strength	High Strength
Cement	242	343	444
Coarse aggregates	605	554	504
Fine aggregates	726	665	605
Fired-clay solid brick aggregates	605	554	504

**Table 3:** Properties of hemp fiber ropes.

Property	Value
Ultimate tensile strength (MPa)	137.4
Nominal diameter (mm)	2.1
Strain (%)	3.5

**Table 4:** Result summary of low-, medium-, and high-strength concrete specimens.

Specimen Name	Peak Stress (MPa)	Increase (%)	Ultimate Strain	Increase (%)
Low-strength concrete				
SR13-LS-CBA-CON	6.13	---	0.0075	---
SR13-LS-CBA-1H	9.78	59	0.0153	104
SR13-LS-CBA-2H	13.33	117	0.0403	438
SR13-LS-CBA-3H	17.24	181	0.0498	564
Medium-strength concrete				
SR13-MS-CBA-CON	12.44	---	0.0067	---
SR13-MS-CBA-1H	17.07	37	0.0132	98
SR13-MS-CBA-2H	22.22	79	0.0237	256
SR13-MS-CBA-3H	26.67	114	0.0333	400
High-strength concrete				
SR13-HS-CBA-CON	15.11	---	0.0060	---
SR13-HS-CBA-1H	20.00	32	0.0117	94
SR13-HS-CBA-2H	24.89	65	0.0138	131
SR13-HS-CBA-3H	29.33	94	0.0175	192

**Table 5:** Existing models for compressive stress and strain.

**Study**	Stress Equation	Strain Equation
Legeron and Paultre 2003 [42]	$f_{cc}=\left[1+2.4{\left(\frac{fl}{f_{co}}\right)}^{0.70}\right]f_{co}$	$\varepsilon_{cc}=\left[1+35{\left(\frac{fl}{f_{co}}\right)}^{1.20}\right]\varepsilon_{co}$
Triantafillou et al. 2006 [44]	$f_{cc}=\left[1+2.79\left(\frac{fl}{f_{co}}\right)\right]f_{co}$	$\varepsilon_{cc}=\left[\varepsilon_{co}+0.082\left(\frac{fl}{f_{co}}\right)\right]$
Akiyama et al. 2010 [46]	$f_{cc}=\left[1+2.4{\left(\frac{fl}{f_{co}}\right)}^{0.647}\right]f_{co}$	$\varepsilon_{cc}=\left[\varepsilon_{co}+0.0766\left(\frac{fl}{f_{co}}\right)\right]$
Shehata et al. 2002 [43]	$f_{cc}=\left[1+0.85\left(\frac{fl}{f_{co}}\right)\right]f_{co}$	$\varepsilon_{cc}=\left[1+13.5\left(\frac{fl}{f_{co}}\right)\right]\varepsilon_{co}$
Cusson and Paultre 1995 [41]	$f_{cc}=\left[1+2.1{\left(\frac{fl}{f_{co}}\right)}^{0.70}\right]f_{co}$	$\varepsilon_{cc}=\left[\varepsilon_{co}+0.21{\left(\frac{fl}{f_{co}}\right)}^{1.7}\right]$
Wang et al. 2012 [45]	$f_{cc}=\left[0.2+3.47{\left(\frac{fl}{f_{co}}\right)}^{0.64}\right]f_{co}$	$\varepsilon_{cc}=\left[2+73.31{\left(\frac{fl}{f_{co}}\right)}^{1.07}\right]\varepsilon_{co}$

**Table 6:** Comparison of experimental and analytical results for low-strength square specimens.

Study	Specimen	*f_cc_* (exp) MPa	*f_cc_* (theo) MPa	*ε_cc_* (exp)	*ε_cc_* (theo)
Legeron and Paultre 2003 [42]	SR13-LS-CBA-1H	9.78	10.15	0.0153	0.0359
	SR13-LS-CBA-2H	13.33	12.67	0.0403	0.0728
	SR13-LS-CBA-3H	17.24	14.81	0.0498	0.1137
Triantafillou et al. 2006 [44]	SR13-LS-CBA-1H	9.78	8.81	0.0153	0.0203
	SR13-LS-CBA-2H	13.33	11.50	0.0403	0.0332
	SR13-LS-CBA-3H	17.24	14.18	0.0498	0.0461
Akiyama et al. 2010 [46]	SR13-LS-CBA-1H	9.78	10.57	0.0153	0.0195
	SR13-LS-CBA-2H	13.33	13.08	0.0403	0.0315
	SR13-LS-CBA-3H	17.24	15.17	0.0498	0.0435
Shehata et al. 2002 [43]	SR13-LS-CBA-1H	9.78	6.95	0.0153	0.0233
	SR13-LS-CBA-2H	13.33	7.76	0.0403	0.0392
	SR13-LS-CBA-3H	17.24	8.58	0.0498	0.0551
Cusson and Paultre 1995 [41]	SR13-LS-CBA-1H	9.78	9.65	0.0153	0.0165
	SR13-LS-CBA-2H	13.33	11.85	0.0403	0.0367
	SR13-LS-CBA-3H	17.24	13.72	0.0498	0.0658
Wang et al. 2012 [45]	SR13-LS-CBA-1H	9.78	7.73	0.0153	0.0907
	SR13-LS-CBA-2H	13.33	11.36	0.0403	0.1740
	SR13-LS-CBA-3H	17.24	14.36	0.0498	0.2605

**Table 7:** Comparison of experimental and analytical results for medium-strength confined square specimens.

Study	Specimen	*f_cc_* (exp) MPa	*f_cc_* (theo) MPa	*ε_cc_* (exp)	*ε_cc_* (theo)
Legeron and Paultre 2003 [42]	SR13-MS-CBA-1H	17.07	17.41	0.0132	0.0175
	SR13-MS-CBA-2H	22.22	20.52	0.0237	0.0316
	SR13-MS-CBA-3H	26.67	23.18	0.0333	0.0473
Triantafillou et al. 2006 [44]	SR13-MS-CBA-1H	17.07	15.12	0.0132	0.0130
	SR13-MS-CBA-2H	22.22	17.81	0.0237	0.0193
	SR13-MS-CBA-3H	26.67	20.50	0.0333	0.0257
Akiyama et al. 2010 [46]	SR13-MS-CBA-1H	17.07	18.14	0.0132	0.0126
	SR13-MS-CBA-2H	22.22	21.36	0.0237	0.0185
	SR13-MS-CBA-3H	26.67	24.04	0.0333	0.0245
Shehata et al. 2002 [43]	SR13-MS-CBA-1H	17.07	13.26	0.0132	0.0137
	SR13-MS-CBA-2H	22.22	14.08	0.0237	0.0207
	SR13-MS-CBA-3H	26.67	14.89	0.0333	0.0277
Cusson and Paultre 1995 [41]	SR13-MS-CBA-1H	17.07	16.80	0.0132	0.0094
	SR13-MS-CBA-2H	22.22	19.51	0.0237	0.0155
	SR13-MS-CBA-3H	26.67	21.83	0.0333	0.0242
Wang et al. 2012 [45]	SR13-MS-CBA-1H	17.07	10.88	0.0132	0.0451
	SR13-MS-CBA-2H	22.22	15.56	0.0237	0.0800
	SR13-MS-CBA-3H	26.67	19.43	0.0333	0.1162

**Table 8:** Comparison of experimental and analytical results for high-strength confined square specimens.

Study	Specimen	*f_cc_* (exp) MPa	*f_cc_* (theo) MPa	*ε_cc_* (exp)	*ε_cc_* (theo)
Legeron and Paultre 2003 [42]	SR13-HS-CBA-1H	20.00	20.38	0.0117	0.0137
	SR13-HS-CBA-2H	24.89	23.68	0.0138	0.0237
	SR13-HS-CBA-3H	29.33	26.49	0.0175	0.0348
Triantafillou et al. 2006 [44]	SR13-HS-CBA-1H	20.00	17.79	0.0117	0.0112
	SR13-HS-CBA-2H	24.89	20.48	0.0138	0.0164
	SR13-HS-CBA-3H	29.33	23.16	0.0175	0.0217
Akiyama et al. 2010 [46]	SR13-HS-CBA-1H	20.00	21.21	0.0117	0.0109
	SR13-HS-CBA-2H	24.89	24.67	0.0138	0.0157
	SR13-HS-CBA-3H	29.33	27.53	0.0175	0.0206
Shehata et al. 2002 [43]	SR13-HS-CBA-1H	20.00	15.93	0.0117	0.0112
	SR13-HS-CBA-2H	24.89	16.74	0.0138	0.0163
	SR13-HS-CBA-3H	29.33	17.56	0.0175	0.0215
Cusson and Paultre 1995 [41]	SR13-HS-CBA-1H	20.00	19.73	0.0117	0.0079
	SR13-HS-CBA-2H	24.89	22.61	0.0138	0.0123
	SR13-HS-CBA-3H	29.33	25.07	0.0175	0.0186
Wang et al. 2012 [45]	SR13-HS-CBA-1H	20.00	12.02	0.0117	0.0351
	SR13-HS-CBA-2H	24.89	17.04	0.0138	0.0605
	SR13-HS-CBA-3H	29.33	21.19	0.0175	0.0868

## Data Availability

The original contributions presented in the study are included in the article, further inquiries can be directed to the corresponding author.

## Appendix A

**Type**	***f_co_* (MPa)**	**Rupture Strength of Fiber (MPa)**	**Fiber Thickness**	**Specimen Size (mm)**	$f_{cc}^{\prime }$ **(MPa)**
Cylinder	16.59	177.5	2.1	150	27.86
Cylinder	16.59	177.5	4.2	150	43.04
Cylinder	16.59	177.5	6.3	150	56.85
Cylinder	16.59	177.5	8.4	150	68.22
Cylinder	16.59	129.3	4.8	150	33.61
Cylinder	16.59	129.3	7.2	150	48.79
Cylinder	16.59	129.3	9.6	150	58.51
Cylinder	16.59	90.8	5.6	150	31.11
Cylinder	16.59	90.8	8.4	150	43.37
Cylinder	16.59	90.8	11.2	150	56.13
Cylinder	24.90	137.4	2.1	150	38.48
Cylinder	24.90	137.4	4.2	150	49.24
Cylinder	24.90	137.4	6.3	150	65.34
Cylinder	16.41	137.4	2.1	150	25.18
Cylinder	16.41	137.4	4.2	150	26.83
Cylinder	16.41	137.4	6.3	150	39.33
Cylinder	12.73	137.4	2.1	150	19.24
Cylinder	12.73	137.4	4.2	150	23.20
Cylinder	12.73	137.4	6.3	150	29.58
Cylinder	13.23	290	1.19	150	25.11
Cylinder	13.23	290	2.38	150	47.79
Cylinder	11.04	290	1.19	150	36.21
Cylinder	11.04	290	2.38	150	47.66
Cylinder	14.83	290	1.19	150	26.33
Cylinder	14.83	290	2.38	150	44.64
Cylinder	19.64	290	1.19	150	36.53
Cylinder	19.64	290	2.38	150	57.91
Cylinder	21.86	290	1.19	150	42.90
Cylinder	21.86	290	2.38	150	63.59
Cylinder	34.48	290	1.19	150	39.27
Cylinder	34.48	290	2.38	150	62.74
Cylinder	13.02	137.4	2.1	150	23.77
Cylinder	13.02	137.4	4.2	150	33.39
Cylinder	13.02	137.4	6.3	150	40.18
Cylinder	24.34	137.4	2.1	150	36.22
Cylinder	24.34	137.4	4.2	150	43.58
Cylinder	24.34	137.4	6.3	150	56.03
Cylinder	19.60	83.58	0.8	150	23.20
Cylinder	19.60	83.58	2.4	150	26.60
Cylinder	19.60	83.58	4	150	31.10
Cylinder	21.00	83.58	0.8	150	23.80
Cylinder	21.00	83.58	2.4	150	28.20
Cylinder	21.00	83.58	4	150	34.30
Cylinder	22.01	80	3	100	37.79
Cylinder	22.01	80	6	100	49.72
Cylinder	22.01	104	3	100	40.40
Cylinder	22.01	104	6	100	59.79
Cylinder	40.75	80	3	100	51.75
Cylinder	40.75	80	6	100	61.08
Cylinder	40.75	104	3	100	57.75
Cylinder	40.75	104	6	100	74.04
Cylinder	58.58	80	3	100	66.87
Cylinder	58.58	80	6	100	69.34
Cylinder	58.58	104	3	100	71.31
Cylinder	58.58	104	6	100	75.35
Cylinder	18.10	104	3	100	39.00
Cylinder	18.10	104	6	100	55.00
Cylinder	18.10	104	9	100	72.00
Cylinder	38.35	104	3	100	53.10
Cylinder	38.35	104	6	100	67.20
Cylinder	38.35	104	9	100	89.00
Cylinder	20.06	174	0.18	100	20.78
Cylinder	20.06	174	0.36	100	22.21
Cylinder	20.06	174	0.53	100	22.69
Cylinder	20.06	174	0.71	100	23.17
Cylinder	20.06	174	0.89	100	25.08
Cylinder	20.06	174	1.07	100	28.66
Cylinder	21.50	179	0.14	100	22.21
Cylinder	21.50	179	0.28	100	24.60
Cylinder	21.50	179	0.42	100	24.84
Cylinder	21.50	179	0.56	100	25.08
Cylinder	21.50	179	0.7	100	25.32
Cylinder	21.50	179	0.84	100	26.99
Cylinder	18.63	104	0.31	100	19.11
Cylinder	18.63	104	0.61	100	19.35
Cylinder	18.63	104	0.92	100	19.58
Cylinder	18.63	104	1.22	100	20.30
Cylinder	18.63	104	1.53	100	20.54
Cylinder	18.63	104	1.83	100	22.69
Cylinder	13.00	250	1.304	100	22.00
Cylinder	18.00	250	1.304	100	25.00
Cylinder	25.00	250	1.304	100	32.00
Cylinder	35.00	250	1.304	100	46.00
SQ1	11.77	177.5	2.1	150	13.64
SQ1	11.77	177.5	4.2	150	16.21
SQ1	11.77	177.5	6.3	150	18.41
SQ1	12.64	177.5	2.1	150	17.00
SQ1	12.64	177.5	4.2	150	18.83
SQ1	12.64	177.5	6.3	150	20.92
SQ1	10.02	177.5	2.1	150	13.08
SQ1	10.02	177.5	4.2	150	13.77
SQ1	10.02	177.5	6.3	150	16.56
SQ1	12.64	177.5	2.1	150	16.54
SQ1	12.64	177.5	4.2	150	20.70
SQ1	12.64	177.5	6.3	150	22.01
SQ1	16.56	177.5	2.1	150	18.74
SQ1	16.56	177.5	4.2	150	21.36
SQ1	16.56	177.5	6.3	150	25.72
SQ1	23.54	177.5	4.2	150	30.51
SQ1	23.54	177.5	6.3	150	36.72
SQ2	10.98	177.5	2.1	150	14.38
SQ2	10.98	177.5	4.2	150	18.04
SQ2	10.98	177.5	6.3	150	21.79
SQ2	14.17	177.5	2.1	150	19.40
SQ2	14.17	177.5	4.2	150	20.81
SQ2	14.17	177.5	6.3	150	27.46
SQ2	11.55	177.5	2.1	150	17.65
SQ2	11.55	177.5	4.2	150	20.49
SQ2	11.55	177.5	6.3	150	24.15
SQ2	18.61	177.5	2.1	150	20.05
SQ2	18.61	177.5	4.2	150	21.14
SQ2	18.61	177.5	6.3	150	23.10
SQ2	21.79	177.5	2.1	150	25.45
SQ2	21.79	177.5	4.2	150	25.89
SQ2	21.79	177.5	6.3	150	28.77
SQ2	20.05	177.5	2.1	150	28.33
SQ2	20.05	177.5	4.2	150	32.04
SQ2	20.05	177.5	6.3	150	30.18
SQ3	11.33	177.5	2.1	150	15.69
SQ3	11.33	177.5	4.2	150	19.40
SQ3	11.33	177.5	6.3	150	23.86
SQ3	15.52	177.5	2.1	150	23.62
SQ3	15.52	177.5	4.2	150	28.24
SQ3	15.52	177.5	6.3	150	32.60
SQ3	10.55	177.5	2.1	150	16.74
SQ3	10.55	177.5	4.2	150	21.23
SQ3	10.55	177.5	6.3	150	26.59
SQ3	13.51	177.5	2.1	150	17.87
SQ3	13.51	177.5	4.2	150	22.01
SQ3	13.51	177.5	6.3	150	24.84
SQ3	16.13	177.5	2.1	150	22.23
SQ3	16.13	177.5	4.2	150	26.59
SQ3	16.13	177.5	6.3	150	27.46
SQ3	23.54	177.5	2.1	150	25.61
SQ3	23.54	177.5	4.2	150	29.64
SQ3	23.54	177.5	6.3	150	31.82
SQ1	16.10	177.5	2.1	150	22.00
SQ1	16.10	177.5	4.2	150	27.40
SQ1	16.10	177.5	6.3	150	33.00
SQ2	15.40	177.5	2.1	150	24.00
SQ2	15.40	177.5	4.2	150	31.60
SQ2	15.40	177.5	6.3	150	38.00
SQ3	14.90	177.5	2.1	150	26.00
SQ3	14.90	177.5	4.2	150	34.50
SQ3	14.90	177.5	6.3	150	42.00
SQ2	28.00	177.5	2.1	150	33.33
SQ2	28.00	177.5	4.2	150	39.11
SQ2	28.00	177.5	6.3	150	42.67
SQ2	18.22	177.5	2.1	150	21.16
SQ2	18.22	177.5	4.2	150	23.11
SQ2	18.22	177.5	6.3	150	24.89
SQ2	17.78	177.5	2.1	150	21.56
SQ2	17.78	177.5	4.2	150	23.11
SQ2	17.78	177.5	6.3	150	24.44
	Note: SQ1: Square specimen with corner radii = 0 mm, SQ2: Square specimen with corner radii = 13 mm, SQ3: Square specimen with corner radii = 26 mm.				
